# Targeted Phototherapy by Niobium Carbide for Mammalian Tumor Models Similar to Humans

**DOI:** 10.3389/fonc.2022.827171

**Published:** 2022-02-10

**Authors:** Zhao Liu, Shan Jiang, Yuhang Tian, Haitao Shang, Kexin Chen, Haoyan Tan, Lei Zhang, Hui Jing, Wen Cheng

**Affiliations:** ^1^ Department of Ultrasound, Harbin Medical University Cancer Hospital, Harbin, China; ^2^ Department of Pathology, Harbin Medical University Cancer Hospital, Harbin, China

**Keywords:** theranostic agent, photothermal/photodynamic co-therapy, macrophage loaded NbC, orthotopic tumor model, CEUS/SWE

## Abstract

**Background:**

In the past few decades, nanomaterial‐mediated phototherapy has gained significant attention as an alternative antitumor strategy. However, its antitumor success is majorly limited to the treatment of subcutaneous tumors in nude mice. In fact, no studies have been previously conducted in this area/field on clinically‐relevant big animal models. Therefore, there is an urgent need to conduct further investigation in a typical big animal model, which is more closely related to the human body.

**Results:**

In this study, niobium carbide (NbC) was selected as a photoactive substance owing to the presence of outstanding near-infrared (NIR) absorption properties, which are responsible for the generation of NIR‐triggered hyperthermia and reactive oxygen species that contribute towards synergetic photothermal and photodynamic effect. Moreover, the present study utilized macrophages as bio‐carrier for the targeted delivery of NbC, wherein phagocytosis by macrophages retained the photothermal/photodynamic effect of NbC. Consequently, macrophage-loaded NbC ensured/allowed complete removal of solid tumors both in nude mice and big animal models involving rabbits. Meanwhile, two‐dimensional ultrasound, shave wave elastography (SWE), and contrast‐enhanced ultrasound (CEUS) were used to monitor physiological evolution in tumor *in vivo* post-treatment, which clearly revealed the occurrence of the photoablation process in tumor and provided a new strategy for the surveillance of tumor in big animal models.

**Conclusion:**

Altogether, the use of a large animal model in this study presented higher clinical significance as compared to previous studies.

## Introduction

Cancer is regarded as a major threat to public health worldwide. The severity of the cancer is highlighted by the absence of any global solution (1). Cancer diseases, such as liver cancer and breast cancer, are the leading causes of death or disability worldwide (2, 3). In clinical practice, the poor therapeutic efficacy of traditional antitumor therapies, like surgery, chemotherapy, or/and radiotherapy, is primarily related to associated side effects and inevitable secondary actions (4, 5). Currently, cancer research is focused on the development of a more efficient way/strategy to improve the accuracy and controllability of oncotherapy. To achieve better treatment outcomes in the patients, there is a need to develop novel therapeutic techniques to surmount the aforementioned issues (6). In recent years, phototherapy, including both photothermal therapy (PTT) and photodynamic therapy (PDT), has emerged as a promising alternative to traditional antitumor therapies, primarily owing to high Spatio-temporal selectivity, low complications, minimal damage, and deeper penetration of near‐infrared (NIR) light (7–10). Phototherapy usually acts *via* light‐triggered photoactive materials, resulting in the generation of local hyperthermia (PTT) or reactive oxygen species (ROS) or PDT to induce cancer cell death (11, 12). Thus, according to this modality, phototherapy could assist in achieving high spatiotemporal accuracy. Although several studies have illustrated the advantages and superiority of PTT or PDT, these two methods are still in the developing stage as the antitumor effect of these methods is restricted to the mice model (13). In a real scenario, the mice model with subcutaneous tumor is quite different from the clinical tumor, in terms of tumor size, depth, and physiological characteristics. Initially, NIR‐mediated phototherapy was developed as a strategy to treat the deep‐site tumor, which relied on the excellent penetration depth of NIR. However, a limited volume of mice and shallow subcutaneous tumors could not examine the true advantages of phototherapy. In fact, the subcutaneous tumor model in mice involves only a thin layer of skin, which is almost equivalent to no barrier for the arrival of NIR. Hence, the currently used mice models fail to evaluate the true efficiency/potential of phototherapy. Before clinical trials, it is important to conduct *in vivo* investigation/assessment in a big animal model, but such studies are seldom done.

Rabbits are one of the most frequently used species in big animal model studies. In fact, the subcutaneous fat layer of rabbits is quite suitable for simulating deep‐site tumors. It has been previously reported that the VX2 carcinoma model in rabbits exhibited great similarities to the human tumor. In fact, VX2 carcinoma could be implanted in several tissues of rabbits and showed significant similarities to the human orthotopic tumor in several aspects, such as vascularization and histological and biological characteristics (14). Furthermore, the thick skin of rabbits and subcutaneous lipid layers are expected to simulate the treatment of deep tumors in humans, to an extreme degree. When compared with the mice model, rabbits are more suitable for the establishment of orthotopic xenograft tumors. In fact, the tumor is expected to be larger in such a case. Altogether, orthotopic tumors in rabbits appear to be more similar to the clinical human tumor (15). However, the fast development of tumors and the high rate of tumor recurrence in rabbits hinder the assessment of phototherapy in a big animal model. Consequently, no reports are currently available on big animal studies in phototherapy. To achieve success in a big animal model, there is a need to enhance antitumor outcomes. In this regard, synergetic PTT and PDT treatment could exert a dual effect on the tumor, enhancing the overall effect of phototherapy. It has been previously reported that PDT action is generally strong in the early stage and becomes weaker with oxygen depletion. In comparison to this, PTT is known to be weaker in early stage and increases with temperature elevation. Consequently, synergetic action of PTT/PDT treatment appears to be promising for effective tumor removal, especially in the case of single-matter-mediated treatment. When compared with PTT/PDT system involving multiple components, it could avoid mutual interference and absorption mismatch between photothermal agent and photosensitizer. Thus, the use of a photoactive material that behaves both as a photothermal agent and photosensitizer should be explored and used for big animal studies.

**Scheme 1:**
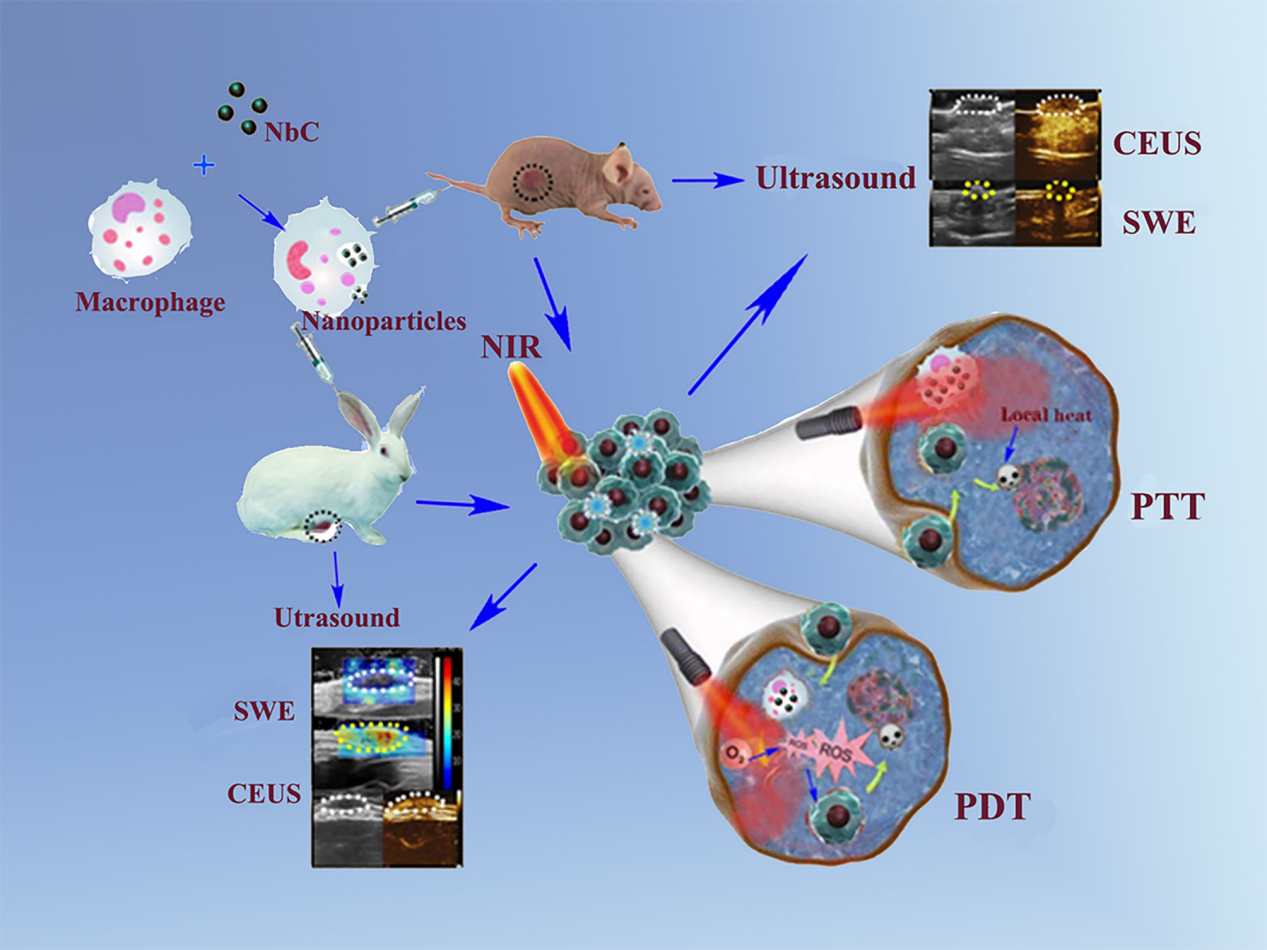
Schematic illustration of NbC@M nanoparticles for targeted phototherapy of nude mice and rabbit VX2 tumor models.

This study presented the use of a novel photoactive material of NbC, which is a NIR‐harvesting material that exhibits both PTT and PDT effects. The study involved the use of cell cargo of macrophages for the loading of NbC nanoparticles. The use of macrophages could target tumors by recognizing cytokines produced/released by cancer cells. Consequently, NbC loaded macrophages (NbC@M) provided/ensured targeted delivery to the tumor site, which was mediated *via* endocytosis by macrophages. In this study, in order to verify the phototherapeutic effect of the NbC@M, we designed the experimental plan using mice with subcutaneous tumors and rabbits with breast cancer of VX2 carcinoma, as shown in **Scheme 1**. The study further used shave wave elastography (SWE) and contrast‐enhanced ultrasound (CEUS) for orthotopic rabbit tumor detection, monitoring, and diagnosis, and thus provided a more convincing evaluation for the treatment outcome of PTT/PDT, which has seldom been investigated in past. Such an evaluation of PTT/PDT outcome holds great clinical significance (16, 17). We expect that NbC@M mediated treatment of the tumor in rabbits could be exploited for clinical application in humans in the future.

## Materials and Methods

### Characterization of NbC Nanoparticles

#### Materials

NbC nanoparticles were obtained from Chaowei Company of Shanghai. The RAW264.7 macrophages, rabbit peripheral blood macrophages and HepG2 cells lines were obtained from the Institute of Cancer Research affiliated to Harbin Medical University. The dulbecco’s modified eagle medium (DMEM, Corning), penicillin/streptomycin (Corning), phosphate buffer saline (PBS), fetal bovine serum (FBS, Gibco) and Trypsin-EDTA solution were purchased from Harbin Shengze Biotechnology Co. Ltd. Calcein-AM, propidium iodide (PI), and 2-(2-methoxy-4-nitrophenyl)-3-(4-nitrophenyl)-5-(2, 4-disulfophenyl)-2H-tetrazolium and monosodium salt (Cell Counting Kit-8, CCK-8) were purchased from Beyotime Biotechnology Co. Ltd. 1, 3- diphenylisobenzofuran (DPBF) and 2’, 7’- dichlorodihydrofluorescein diacetate (DCFH-DA) were obtained from Sigma-Aldrich.

#### Characterization

The morphologies of NbC were observed by TEM (JEOL JEM-2100, Japan). The size distribution analysis was conducted on Zetasizer Nano S90 (Malvern Panalytical, UK). The crystal phase was tested by XRD (Shimadzu XD-D1). The composition and chemical valence of NbC were measured by XPS spectra (PerkinElmer PHI 5600). UV-vis-NIR absorptive spectra of samples were performed on a spectrophotometer (U-4100, Hitachi, Japan). The concentration of Nb element within cells was analyzed by inductively coupled plasma (ICP) atomic emission spectrometer (8300, PerkinElmer, USA). Two-dimensional ultrasound (2D US), Color Doppler Flow Imaging (CDFI) and contrast-enhanced ultrasound (CEUS) scans were performed using MyLab twice system (Esaote SpA, Florence, Italy) with LA523 probe. All the data of shave wave elastography (SWE) were recorded by a real-time US device (Aixplorer; SuperSonic Imagine, Aix-en-Provence, France) with 4-15 MHz liner transducer.

#### Photothermal Test

0.5 mL NbC aqueous dispersion of various concentrations was irradiated with 808 nm NIR laser (1.0 W/cm^2^) for 10 minutes. The changes of solution temperature were collected by a NIR camera (E6, FL-IR Systems, Inc, USA). The photothermal conversion efficiency was calculated according to the photoheating curve of the equivalent NbC.

### Characterization of NbC Loaded Macrophages

#### Cell Lines and Cell Culture

The RAW264.7 macrophages, rabbit peripheral blood macrophages and HepG2 cells lines were cultured in DMEM supplemented with 10% FBS and 1% penicillin/streptomycin in a humidified atmosphere of 5% CO_2_ at 37°C. Hemocytometer (Bürker-Türk, Wertheim, Germany) was used for the total cell counting.

#### Preparation of the Macrophage-Loaded NbC Nanoparticles

The RAW264.7 macrophages and rabbit peripheral blood macrophages were incubated with DMEM until the cell coverage reached about 80% ~ 90%. Then, the stale DMEM was removed and the fresh DMEM supplemented with various concentrations of NbC (0.125, 0.25, 0.5, 1 and 2 mg/mL) and incubated for varied time (2, 4, 6, 12 and 24 h). After incubation, the supernatant was removed and macrophage-loaded NbC nanoparticles were washed with PBS twice. The macrophages were observed with inverted biological microscope (Olympus CKX41). ICP analysis was used to quantify the uptake amount of Nb content in the macrophage cells.

#### Detection of ROS

DPBF solution with NbC or NbC@M was respectively placed in a quartz tube, and then irradiated with 808 nm NIR laser (1 W/cm^2^) for 10, 20, 40 and 60 min. Then, absorbance at 420 nm was determined by U-4100 spectrophotometer. In order to detect intracellular reactive oxygen species (ROS), HepG2 cells were divided into five groups, including untreated control (group 1), cells incubated with NbC@M (0.2 mg/mL) for 4 h (group 2), 808 nm NIR laser (1 W/cm^2^) irradiated cells (group 3), positive control with H_2_O_2_ (200 µL, 50 × 10^-3^ M) at 37 °C for 1 h (group 4), NbC@M mediated phototherapy (group 5). After the treatments, the cells treated in different ways were stained with DCFH-DA (50 µL, 50 × 10^-3^ M) for 1 h and then imaged on inverted biological microscope.

#### Cytotoxicity Assay

The standard CCK-8 assay was used to evaluate the viability of cells. HepG2 cells (8×10^3^ cells/well) were seeded into 96-well plates and cultured for 12 h. NbC@M solutions of different concentrations were then added into above wells and incubated for another 12 or 24 h, respectively. After washed by PBS solution for three times, 10 µL CCK-8 was added to the samples and incubated for another 1 h. The absorbance at 450 nm was then recorded on a microplate reader (BioTek, Winooski, VT, USA). The CCK-8 method was also applied for analyzing the therapeutic effect of *in vitro* PDT, PTT, and PDT+PTT experiments. Similarly, HepG2 cells (8×10^3^ cells per well) in each group were cultured in 96-well plates for 12 h. Then, 100 µL NbC@M (500 µg/mL) were added into each well and incubated for another 12 h, respectively. All groups were subjected to 808 nm NIR irradiation except control group. Additional operations are needed to handle PDT and PTT group. The process of PDT should be set in an ice bath, while sodium azide (50 µL, 1×10^-5^ M) was added into the medium of PTT group before NIR irradiation.

### Phototherapeutic Effects of NbC@M *In Vitro*

To evaluate the phototherapeutic effects at the cellular level, HepG2 cells (3×10^5^ cell/dish) were cultured with DMEM medium containing NbC or NbC@M (2 mL, 250 µg/mL) in 35 mm culture dishes for 6 h. Redundant NbC or NbC@M was removed by washing with PBS and then 500 µL fresh DMEM medium was added into each dish. Then, above resultant cells were irradiated with 808 nm NIR laser (1 W/cm^2^) for 2, 5, and 10 min. Several groups were designed as controls, including untreated cells, cells only irradiated by 808 nm NIR laser (1 W/cm^2^), and incubated with NbC or NbC@M. Finally, dead and live cells were labeled by Calcein-AM (1 µg/mL, 200 µL) and PI (20 µg/mL, 200 µL) for 20 min, and then washed with PBS. The fluorescence visualization images were imaged on Olympus BX53 microscope.

### Phototherapeutic Effect of NbC@M in Nude Mice and Rabbits Tumor Model

#### *In Vivo* Phototherapy on Mice

All experimental female Balb/c nude mice were purchased from Beijing Vital River Laboratory Animal Technology Co., Ltd. HepG2 cells were digested and dispersed in DMEM to form a uniform suspension, which were then injected into the subcutaneous tissue of the upper left hind leg of nude mice to establish tumor-bearing mice model (tumor size is about 150 mm^3^). Then, tumor-bearing mice were randomly divided into four groups (n = 5 for each group): group 1 (100 µL PBS was injected intravenously as control), group 2 (808 nm NIR irradiation for 10 min, 1 W/cm^2^), group 3 (NbC@M was injected intravenously) and group 4 (intravenous injection of NbC@M plus 808 nm NIR irradiation for 10 min). NbC@M (100 µL) was injected intravenously into nude mice at a concentration of 1 mg/mL in the group 3 and 4, respectively. In the group 2 and 4, 808 nm NIR irradiation (1 W/cm^2^) was required to irradiate the tumor area for 10 min. Notably, the mice in the group 4 should be irradiated at 12 hours post injection of NbC@M. The change of temperature was monitored and recorded every 30 s by an FL-IR System E6 infrared camera. The data of tumor size and body weight of nude mice were recorded in detail for 14 days. The tumor volume was calculated as *V* = length × width^2^/2. Relative body weight was designed as dividing instant weight by initial weight (W/W_0_), and similarly relative tumor volume was defined as V/V_0_. All the animal experiments were approved by the criteria of the National Regulation of China for Care and the Ethics Research Committee of Harbin Medical University Cancer Hospital.

#### Rabbit VX2 Breast Xenograft Model

All of the female New Zealand white rabbits (12~14 weeks old, 2.5 ~ 3.0 kg) were purchased from animal laboratory of the Second Affiliated Hospital of Harbin Medical University. The VX2 tumors were surgically implanted into the left second nipple areola edge for each rabbit. The VX2 tumor mass was obtained from the hind limb of a donor rabbit and minced approximately into 1 mm^3^ fragments, which were dispersed in normal saline to form a suspension. Extract a small amount of suspension and ensure that at least two VX2 tumor fragments were injected into the designated location. Ten days after tumor implantation, the tumors should be observed until the tumors’ volume reached up to about 300 ~ 500 mm^3^.

#### *In Vivo* Phototherapy on Rabbits

Twelve VX2 tumor-bearing rabbits were divided into four groups randomly (n = 3 for each group), including injection of PBS (1 mL) as control, receiving only NIR irradiation, receiving only injection of NbC@M (1 mL, 1 mg/mL), and receiving NIR irradiation at 8 h post-injection of NbC@M (1mL, 1 mg/mL). For the real-time thermal imaging, tumor regions were irradiated by an 808 nm laser with a power density of 2 W/cm^2^ for 20 min, and then monitored with an infrared imaging device (FL-IR System E6). All injections of PBS or NbC@M are performed through the posterior auricular vein.

#### Ultrasound on Mice and Rabbits

Ultrasound tests as a general term, including two-dimensional ultrasound (2D US), Color Doppler Flow Imaging (CDFI), SWE and CEUS, were completed by two radiologists with more than five years of experience. After confirming the tumor area and applying enough ultrasonic coupling gel during SWE process, the probe was kept in a stable position with no pressure for about 3 seconds and was vertical to better reduce compression artifacts. A suitable region of interest (ROI) and scale ruler were identical for the elastographic quantitative analysis (SWE_max_ and SWE_mean_). CEUS examinations were performed with intravenous vein injection of SonoVue (Bracco SpA, Milan, Italy) followed by a saline flush. 100 µL SonoVue was injected into the caudal vein for mice, while 1 mL SonoVue was injected into the posterior auricular vein for rabbits. Video clips of the examinations, including the process of enhancement and washout, were immediately recorded over a time period of 1 min after injection of SonoVue.

#### Histological and Blood Biochemistry Analysis

To perform histological analysis, all the mice and rabbits were sacrificed at 14^th^ day after treatment. Hematoxylin and eosin (H&E) staining of major organs including heart, liver, spleen, lung, kidney was done for histopathologic analysis. Slices were observed by a digital microscope (magnification: ×100; DM3000; Leica, Germany). The blood (20 μL) of mice were collected and tested at 14^th^ day after treatment by automatic blood analyzer (HF-3800).

## Results and Discussion

### Characterization of NbC Nanoparticles

**Figure 1:**
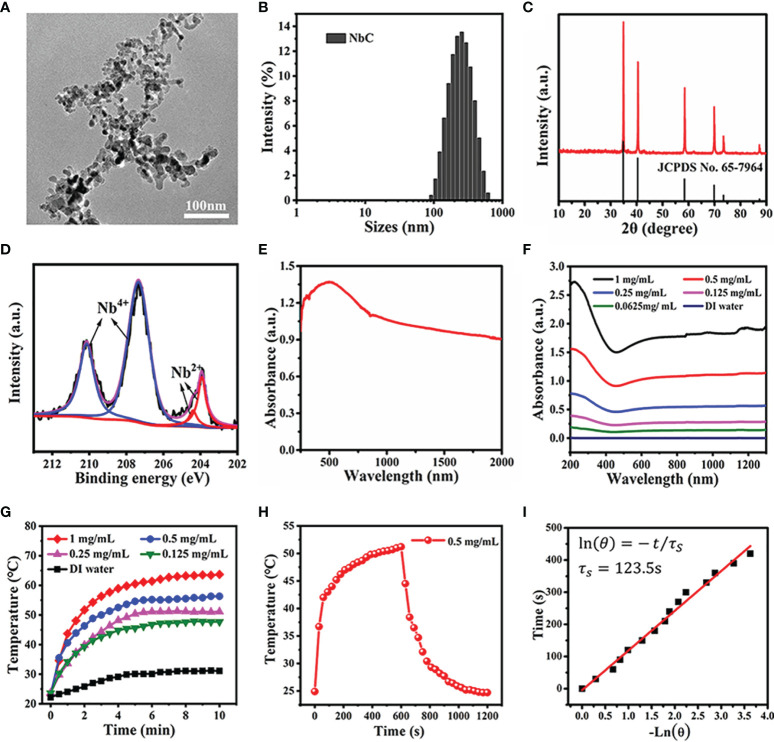
Characterization of the NbC nanoparticles. **(A)** TEM image. **(B)** The hydrodynamic size of NbC measured by the dynamic light scattering (DLS). **(C)** X-ray diffraction pattern of NbC. **(D)** XPS spectra of Nb 3d orbit). **(E)** Powder absorbance of NbC. **(F)** Absorbance of NbC aqueous dispersion. **(G)** Photothermal heating curves of the NbC aqueous dispersion with different concentrations under NIR laser irradiation (808nm, 1W/cm^2^). **(H)** Heating and cooling curves of NbC dispersion. **(I)** Plot of time versus the negative natural logarithm of the driving force temperature.

In this study, NbC nanoparticles were employed as photoactive material for phototherapy. The morphological features of these nanoparticles were assessed using transmission electron microscopy (TEM). As shown in **Figure 1A**, TEM micrographs revealed that NbC nanoparticles were ~10–30 nm in size. However, the aggregation of particles appeared to be a concern. Dynamic light scattering (DLS) results showed that the average hydrodynamic size of NbC nanoparticle was 255 nm (**Figure 1B**), which was bigger as compared to TEM measurements. This further verified the aggregation of the nanoparticles. The crystal phase and crystallinity of the powdered sample were characterized by X‐ray diffraction (XRD). As shown in **Figure 1C**, all the diffractive peaks were consistent with JCPDS No. 65‐7964 of NbC species, and no impurities were reported. XPS survey on core‐level of NbC’s 3d orbit revealed the presence of two spin‐orbit coupling doublets, corresponding to Nb^4+^ and Nb^2+^ ions, after fitting (**Figure 1D**). The aforementioned results further verified the purity of NbC nanoparticles.

Following this, the optical absorbance of NbC nanoparticles was examined. The results of the same are shown in **Figures 1E, F**. Spectroscopy measurements showed that powdered NbC exhibited strong and wide photoabsorption in the range of 300–2000 nm. Consequently, powdered NbC covered the biological window of 700–1200 nm that is normally used for phototherapy (**Figure 1E**). Meanwhile, the absorbance of an aqueous solution of NbC presented a similar wide optical absorption band, and the absorbance was positively correlated to the concentration of NbC (**Figure 1F**). Next, the photothermal conversion effect was assessed. As shown in **Figure 1G**, aqueous solution of various concentrations of NbC exhibited a sharp temperature increment under 808 nm of NIR irradiation, with a safe power density of 1 W/cm^2^, when measured in contrast to deionized (DI) water control that usually involves a slight temperature elevation only. Thus, the higher the concentration of the sample, the higher was the temperature elevation. To measure photothermal conversion efficiency, the curve of temperature change was assessed for 20 min (**Figure 1H** **)**, which involved 808 nm (1W/cm^2^) irradiation for 10 mins and natural cooling for the next 10 mins. According to the previously reported method (18, 19), the value of photothermal conversion efficiency (η) for NbC was evaluated to be 47.5% (**Figure 1I**), which was much higher as compared to the most commonly used PTT agent. This evidence indicated that NbC exhibited excellent photothermal properties, and thus could be used for PTT treatment of tumors.

### Characterization of NbC Loaded Macrophages

**Figure 2:**
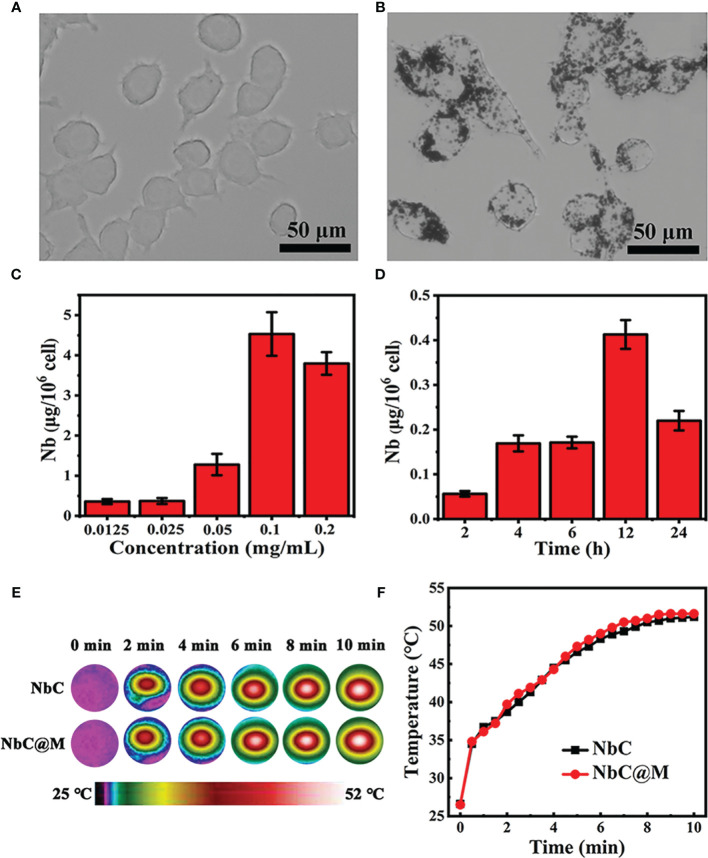
**(A)** Micrograph of macrophages and **(B)** NbC nanoparticles loaded macrophages. The influence of **(C)** concentration and **(D)** incubation time on intracellular NbC content in macrophage by ICP-MS measurement. **(E)** Thermography image and corresponding **(F)** photo-heating curve of the equivalent NbC and macrophage-engulfed NbC (NbC@M).

In this study, macrophages were used as bio‐cargo for targeted delivery of NbC to the tumor site. First, the payload capability of macrophages towards NbC, mediated *via* endocytosis, was assessed. Briefly, NbC nanoparticles were incubated with macrophages to form an NbC@M complex, under different incubation times and NbC concentrations. Following this, uptake of NbC by macrophages was studied as a function of incubation time and concentration of NbC using inductively coupled plasma mass spectrometry (ICP‐MS) measurement. The micrographs for macrophages before and after loading of NbC are shown in **Figures 2A, B**. These micrographs clearly demonstrated the endocytosis of NbC by macrophages. The quantitative assessment further confirmed that optimal incubation conditions involved incubation time of 12 h and NbC concentration of 0.1 mg/mL (**Figures 2C, D** **)**, wherein macrophages exhibited the best performance in terms of endocytosis. Interestingly, when the concentration of NbC was too high, the intracellular content of NbC was significantly reduced, which might be contributed by surpassing the maximum amount of macrophage endocytosis that ultimately leads to cell death. Altogether, macrophages acted as an ideal drug carrier. The study next assessed whether macrophage loading affects the photothermal behavior of the nanoparticles. As shown in **Figures 2E, F**, no significant differences were recorded in the temperature rising trends for equivalent NbC and macrophage‐engulfed NbC (NbC@M), which indicated that endocytosis of the nanoparticles by macrophages did not confer any effect on the photothermal capacity of NbC.

### Phototherapeutic Effects of NbC@M *In Vitro*

**Figure 3:**
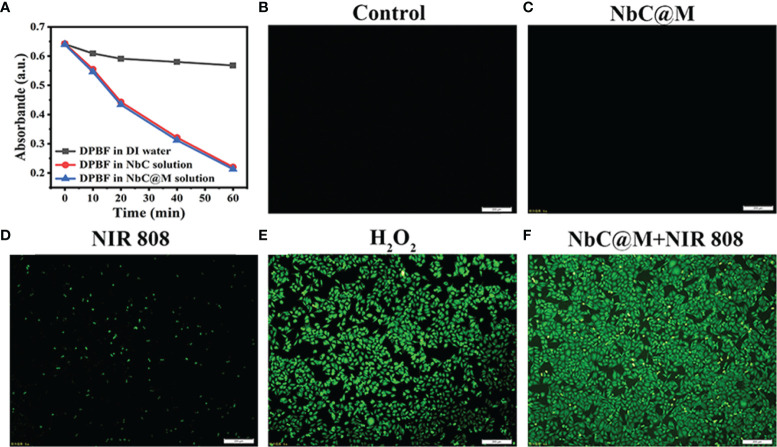
Detection of ROS generation. **(A)** Degradation curve of DPBF. Fluorescence microscopy images of ROS level for **(B)** cells without any treatment as a negative control, **(C)** cells incubated with NbC@M, **(D)** cells received NIR irradiation, **(E)** H_2_O_2_ treated cells as a positive control, **(F)** cells treated with NbC@M and NIR irradiation (808 nm, 1W/cm^2^, 10 min; scale bar = 200μm).

The present study aimed to use NbC both as a photothermal agent for PTT and a photosensitizer to generate reactive oxygen species (ROS) for the PDT (20). Two methods were used to detect the production of ROS. The first method involved the use of a DPBF probe, which could be decomposed by ROS, resulting in a decrement in its typical absorption peak at 420 nm. Thus, for the evaluation of ROS levels, loss of DPBF absorption could be measured using spectrophotometry. As shown in **Figure 3A**, NbC@M, and NbC could mediate faster and higher DPBF loss as compared to the control group, which indicated significant production of ROS under the effect of 808 nm NIR irradiation. Moreover, the degradation curve of DPBF for NbC@M and NbC were nearly similar/same, which further suggested that macrophage loading conferred no detrimental effect on photosensitive properties of NbC. Following this, another probe, 2’,7’‐dichlorodihydrofluorescein diacetate (DCFH‐DA) was employed for the detection of intracellular ROS levels. Principally, ROS can oxidize non‐fluorescent DCFH‐DA to produce green fluorescent 2’,7’‐dichloroflorescein (DCF), which can be used to determine the levels of ROS in cells in terms of visual fluorescence. When compared with other control groups, bright green fluorescence was observed in the NIR‐excited NbC@M group, which was found to be similar to the fluorescence observed in the positive control group that comprised of H_2_O_2_ treated samples. These results indicated the generation of ROS within the cells (**Figures 3B–F**). Therefore, the phagocytosis by macrophages incurred no effect on PTT and PDT generated by NbC@M, under NIR irradiation.

**Figure 4:**
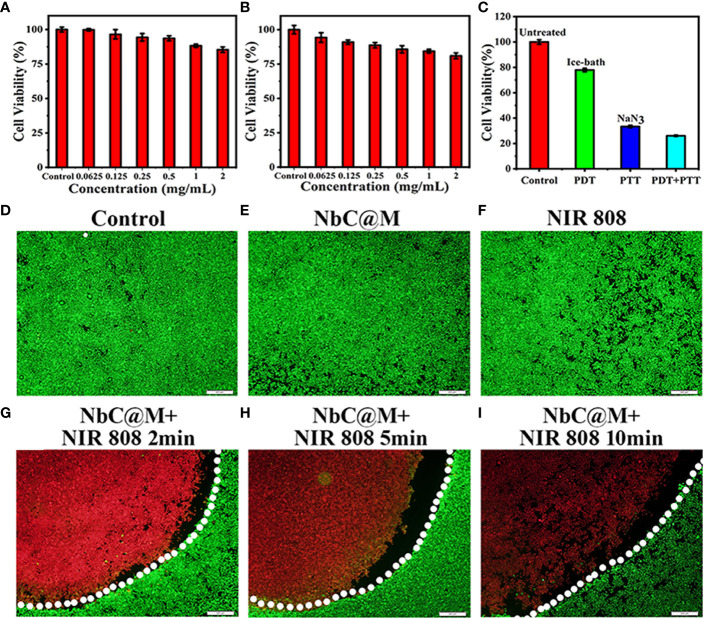
*In vitro* cell cytotoxicity assay and phototherapeutic effect of NbC@M nanoparticles. Relative cell viability of HepG2 cells treated with different concentrations of NbC@M nanoparticles for 12h **(A)** and 24h **(B)**. **(C)** Relative cell viability after different treatments with NbC@M nanoparticles. **(D–I)** Fluorescence images of Live/dead HepG2 cells after receiving different treatments (808 nm, 1W/cm^2^, 10 min; scale bar = 500μm).

The biocompatibility of NbC@M was examined using CCK‐8 assay, wherein low cytotoxicity of NbC@M was recorded in HepG2 cells without irradiation. The cell viability was recorded to be over 85% and 80% after 12 h and 24 h of incubation, respectively, even at the concentration of 2 mg/mL (**Figures 4A, B** **)**. Following this, *in vitro* phototherapy evaluation was performed under NIR irradiation. In the view of the dual‐role of NbC that allows simultaneous generation of ROS and hyperthermia, the use of NbC nanoparticles might provide both PDT and PTT effects to the cancer cells. For investigating a single contribution from PDT or PTT, an ice bath or ROS quencher NaN_3_ was introduced to eliminate heat or ROS, respectively. Following this, the CCK‐8 method was used to analyze the cell viability of HepG2 after different treatments. As shown in **Figure 4C**, the single role of PDT resulted in 22% of cancer cell death, while PTT incurred 64% of cancer cell death. In comparison to this, the combination of PDT/PTT (without the introduction of NaN_3_ and ice bath) showed the best cancer cell killing effect, wherein 74% cell death was recorded. At the same time, the killing effect of PTT was found to be much better as compared to PDT. Thus, the aforementioned results verified the synergetic action of PDT and PTT for the ablation of cancer. Besides this, a fluorescence staining method involving Calcein AM and propidium Iodide (PI) was used to visually evaluate the NbC@M‐triggered phototherapy effect. In particular, live cells give green fluorescence when stained using Calcein AM, whereas PI staining results in red staining in dead cells. As shown in fluorescence microscopic images (**Figures 4D–F**, **S1A–C**), control group, NbC@M treated cells, NbC treated cells, and 808 nm NIR irradiated cells exhibited green fluorescence only, indicating that neither NIR irradiation nor NbC@M killed HepG2 cells. In comparison to this, macroscopic cancer cell death was confirmed in NbC@M or NbC meditated phototherapy groups, as indicated by the presence of distinct red fluorescence (**Figures 4G–I**, **S1D–F**). Moreover, the number of dead cells positively correlated with the duration of irradiation. In this study, NbC played a major role in the killing of tumor cells, regardless of the loading in macrophages. Altogether, these results suggested that NbC@M acted as an effective NIR‐triggered synergetic PTT/PDT agent for cancer treatment.

### Phototherapeutic Effect of NbC@M in Nude Mice Tumor Model

**Figure 5:**
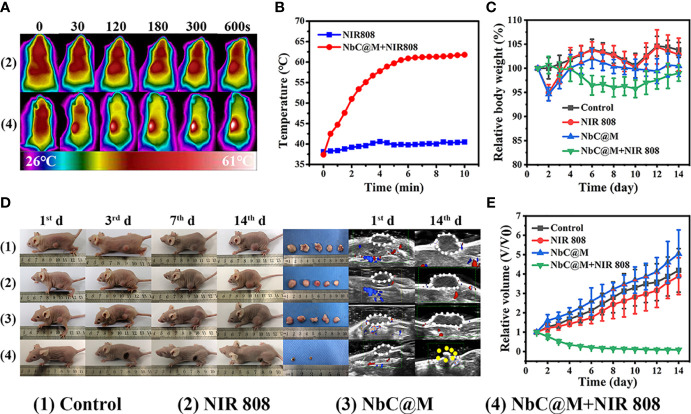
*In vivo* phototherapy study. **(A)** Photothermal images of HepG2 tumor-bearing mice under NIR irradiation (808 nm, 1W/cm^2^) and **(B)** corresponding temperature variation at tumor sites under NIR irradiation. **(C)** Quantitative measurements of relative body weight after different treatments during 14 days. **(D)** Representative photographs of mice, tumors and ultrasound image during the treatments. **(E)** Relative tumor volume after different treatments during 14 days.

**Figure 6:**
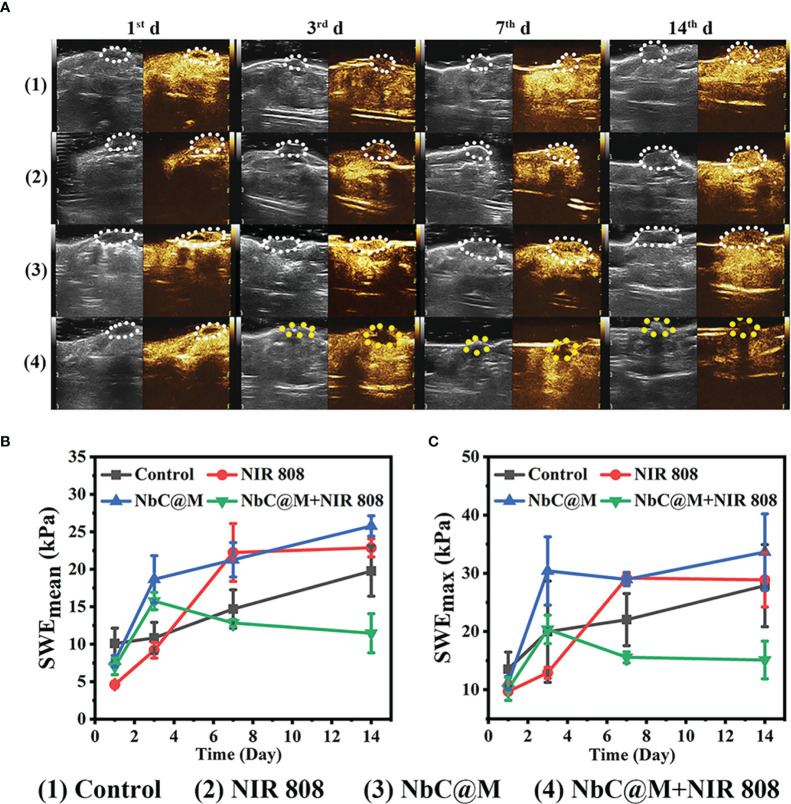
Contrast-enhanced ultrasound (CEUS) and shave wave elastography (SWE) observation on HepG2 tumor-bearing mice after different treatments. **(A)** CEUS detection of tumor internal changes during 14 days post treatment. The changing trend in terms of mean **(B)** and maximum **(C)** values of the SWE at the tumor site during 14 days post treatment.

The excellent results for phototherapeutic properties of NbC@M *in vitro* encouraged the assessment of NbC@M *in vivo*, wherein HepG2 tumor-bearing nude mice were first used as a typical animal model. Briefly, 20 nude mice were randomly divided into four groups, namely the control group (PBS was only injected intravenously), mice treated with 808 nm NIR irradiation, mice treated with NbC@M injection intravenously, and mice treated with NbC@M + 808 nm NIR irradiation. An infrared thermal camera was used to record temperature changes at the tumor location. As shown in **Figure 5A**, tumor site in group 4 exhibited a rapid increase in temperature from 37.4°C to 61.7°C, while group 2 displayed only a slight increase in temperature from 38.1°C to 40.5°C (**Figure 5B**). The only difference between groups 2 and 4 was the presence or absence of NbC@M, which verified the *in vivo* photothermal ability of NbC@M. Following the application of phototherapy, vernier caliper and electronic autobalance were used to monitor changes in tumor volume and weight of mice for 14 consecutive days. Meanwhile, 2D US, CDFI, CEUS, and SWE images and photographs for mice in all groups were obtained on the 1^st^, 3^rd^, 7^th^, and 14^th^ day, respectively. During this period, no significant differences were recorded in the bodyweight among different groups of mice (**Figure 5C**). Importantly, it was observed that the volume of tumors in groups 1–3 increased during the study, while the tumors in group 4 were found to shrink gradually until they completely disappeared (**Figures 5D, E**, **S2**), leaving only scars on the skin. The scabs without blood supply were observed in group 4 after treatment, while tumors in the other three groups were not destroyed and retained blood vessels, and thus high blood perfusion could be seen by CEUS (white dotted line outline, **Figure 6A**) in these three groups. In contrast to this, scabs in group 4 showed no blood perfusion area (yellow dotted line outline, **Figure 6A**). In addition to blood perfusion, SWE value for the tumor site in all groups changed continuously over time. The SWE values for tumors in group 1, group 2, and group 3 showed a continuous upward trend, both in terms of mean and maximum values. On the contrary, SWE values for scabs in group 4 increased significantly on the 3^rd^ day, but decreased on the 7^th^ and 14^th^ day, as shown in **Figures 6B, C**, **S3**. These results demonstrated that the tumors in the three control groups progressed continuously, and the use of 808 nm laser irradiation or NbC@M alone could not ablate the tumors. In comparison to this effective antitumor treatment was observed in the case of NbC@M mediated PTT/PDT. The results for CEUS and SWE showed that when compared with the control group, the tumor area got cured and scabbed, and gradually improved. In fact, no blood perfusion and low SWE value were observed in this group. To evaluate *in vivo* toxicity of NbC@M, the main organs (heart, liver, spleen, lung, and kidney) and blood were extracted from mice in groups 1–4 on the 14^th^ day. The results for hematoxylin-eosin (H&E) staining showed no obvious pathological changes. Meanwhile, the hemanalysis indicated no blood abnormalities in groups 1–4 (**Figures S4**, **S5**), which indicated biosafety of NbC@M for phototherapy.

### Phototherapeutic Effects of NbC@M in Rabbits Tumor Model

**Figure 7:**
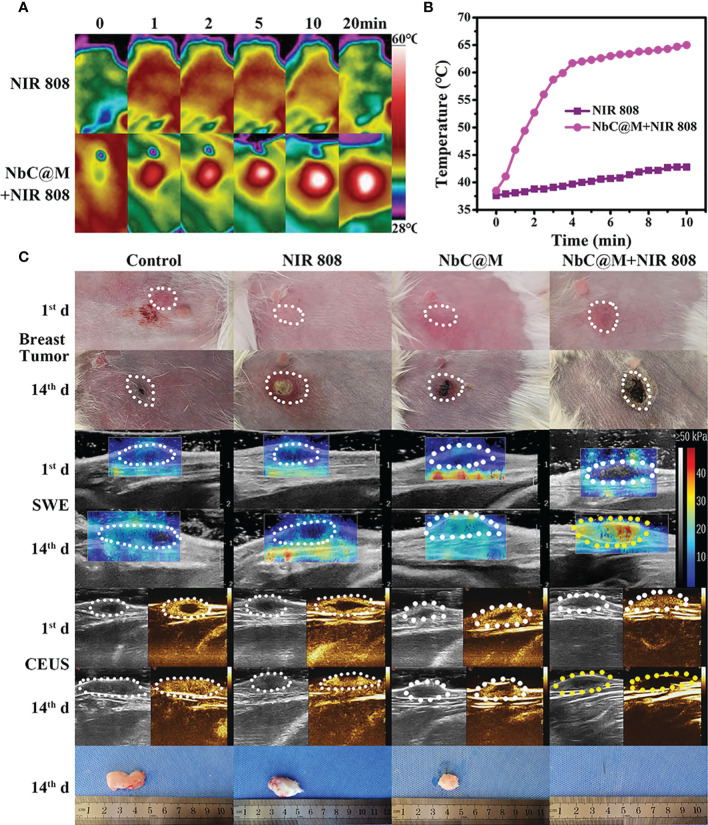
*In vivo* phototherapy study of rabbits. **(A)** Photothermal images and corresponding **(B)** temperature variation at tumor site under NIR irradiation. **(C)** Representative photographs of tumor sites on rabbits and tumors, SWE and CEUS images of tumor site.

In this study, rabbit VX2 carcinoma, a squamous epithelioid carcinoma with rapid growth, was used to develop breast tumors in rabbits, primarily owing to the presence of a similar tumor microenvironment in rabbits and humans. Similar to the aforementioned nude mice experiment, rabbits were also divided into four groups. The occurrence of thicker skin in rabbits made it difficult to measure tumor volume with vernier calipers, and thus B‐mode ultrasonography was used for volume measurements. As shown in **Figure 7A**, the temperature changes for NIR 808 group and NbC@M + NIR 808 group were found to be similar to that of the nude mice experiment. In sharp contrast to the control group, the temperature of the tumor area increased rapidly up to 65°C in NbC@M + NIR 808 group (**Figure 7B**). And the penetration distance of NIR 880 nm laser is 10mm, which can meet the needs of this study (21, 22). It is well known that insufficient internal blood supply might lead to the appearance of necrotic areas in the tumors, which could break through the skin and drain pus (**Figure 7C**). Hence, it appeared that the size of the tumors did not increase, but in reality, these tumors were rapidly progressing. Over time, SWE values for the tumors in control, NIR 808, and NbC@M groups increased only slightly, while SWE values for the tumors in NbC@M + NIR 808 group increased significantly, primarily due to the presence of scab (**Figure S6**). Moreover, the expanding necrotic areas in control, NIR 808, and NbC@M groups were characterized by abundant blood supply within the tumors and no blood supply in the necrotic areas, as observed by CEUS. The tumor areas in NbC@M + NIR 808 group were not enhanced, which was different when compared with the other three groups. The mutual confirmation of SWE and CEUS results indicated that the tumors were completely cured by the PDT/PTT effects of NbC@M. Furthermore, no significant pathological changes were observed in all groups of rabbits (**Figure S7**), indicating negligible biological toxicity of NbC@M. Thus, the present study demonstrated the successful application of phototherapy in a big animal model, involving the use of rabbits.

## Conclusion

Altogether, this study presented the application of phototherapy, both in mice models and large animal models of rabbits, in a more clinically relevant manner. In particular, the study demonstrated that NbC photoactive material exhibited excellent NIR‐harvesting performance, particularly in terms of hyperthermia and ROS generation. In fact, significant photothermal and photodynamic capabilities of NbC were retained even after loading within macrophages, which served as a carrier for the targeted enrichment in the tumor. The SWE and CEUS results showed that the effect of the NbC@M‐mediated PTT/PDT method successfully cured subcutaneous tumors in mice and orthotopic tumors in rabbits. In addition to this, NbC@M exhibited negligible hematotoxicity and systemic toxicity. Therefore, this study provided evidence for the suitability of NbC@M‐mediated PTT/PDT to be explored for clinical treatment.

## Data Availability Statement

The original contributions presented in the study are included in the article/**Supplementary Material**. Further inquiries can be directed to the corresponding author.

## Ethics Statement

The animal study was reviewed and approved by the institutional research ethics committee of Harbin Medical University.

## Author Contributions

ZL worked at concept design, experiment arrangement and manuscript drafting. YT carried out the synthesis and characterization of theranostic agent. SJ, HS, and HT participated in the cell mice and rabbit experiment. KC was responsible for diagnosis and analysis of pathological section. ZL and LZ were in charge of summarizing and analyzing experimental data. HJ and WC joined the study’s design and performed review and editing. All authors read and approved the final manuscript.

## Funding

This work was funded by the National Natural Science Foundation of China (81873900 and 81801709), Key projects of Natural Science Foundation of Heilongjiang Province (ZD2021H005), Science Foundation of Health Commission of Heilongjiang Province (2019052), Innovative scientific research funding project of Harbin Medical University (2020-KYYWF-1471), HAI YAN Science Foundation of Harbin Medical University Cancer Hospital (grant no. JJQN2018-18, JJQN2018-15, JJQN2020-21 and JJQN2021-08).

## Conflict of Interest

The authors declare that the research was conducted in the absence of any commercial or financial relationships that could be construed as a potential conflict of interest.

## Publisher’s Note

All claims expressed in this article are solely those of the authors and do not necessarily represent those of their affiliated organizations, or those of the publisher, the editors and the reviewers. Any product that may be evaluated in this article, or claim that may be made by its manufacturer, is not guaranteed or endorsed by the publisher.
