# Supplementary material for: Targeted Phototherapy by Niobium Carbide for Mammalian Tumor Models Similar to Humans
Source: Front Oncol. 2022 Feb 10;12:827171. doi: 10.3389/fonc.2022.827171 (PMC8866440; doi:10.3389/fonc.2022.827171)
Supplement: Supplementary file 1 [file DataSheet_1.docx]

**Supporting information**

**Targeted Phototherapy by Niobium Carbide for Mammalian Tumor Models Similar to Humans**

Zhao Liu^1^, Shan Jiang^1^, Yuhang Tian^1^, Haitao Shang^1^, Kexin Chen^2^, Haoyan Tan^1^, Lei Zhang^1^, Hui Jing^1^, Wen Cheng^1^

1 Department of Ultrasound, Harbin Medical University Cancer Hospital, 150 Haping Road, Nangang District, Harbin, 150081, China

2 Department of Pathology, Harbin Medical University Cancer Hospital, 150 Haping Road, Harbin, 150081, China

Corresponding author

Email: hrbchengwen@163.com
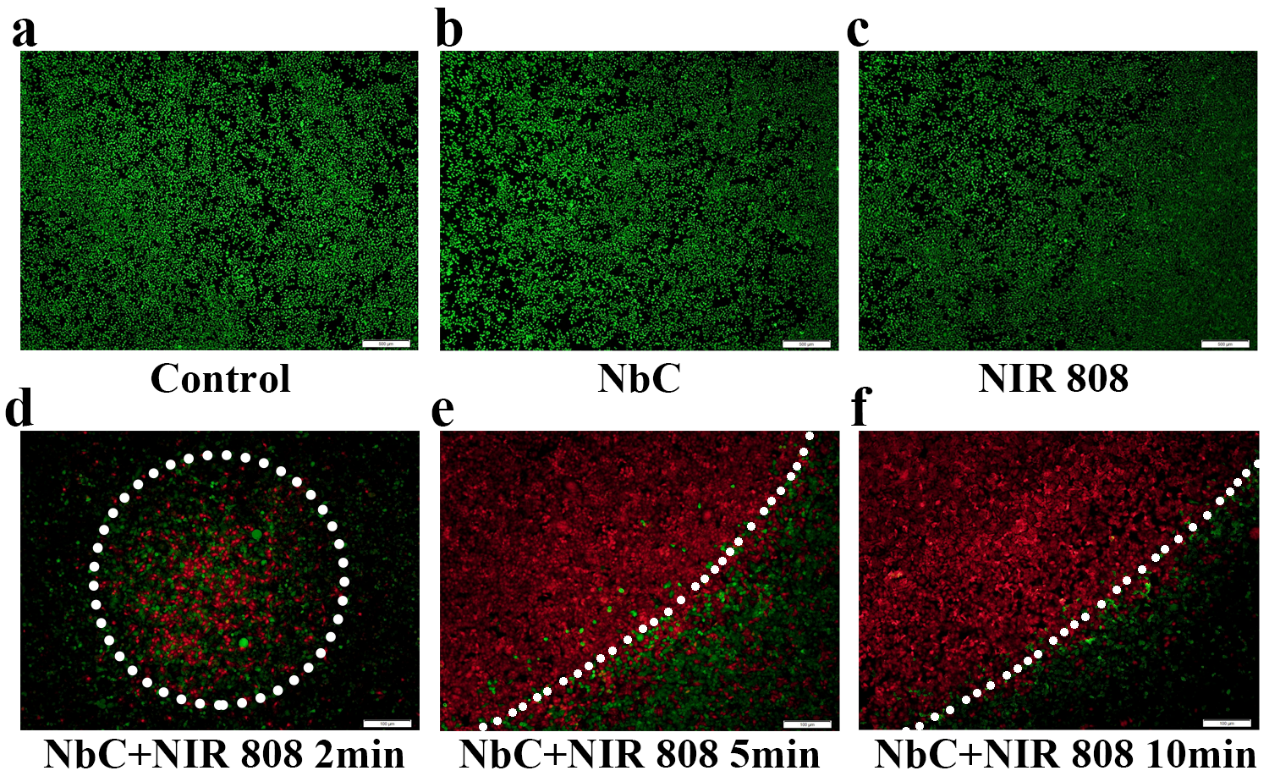


**Figure. S1** In vitro cell phototherapeutic eﬀect of NbCnanoparticles. (a-f) Fluorescence images of Living/dead HepG2 cells after receiving diﬀerent treatments (808 nm, 1W/cm^2^, 10 min; a-c: scale bar = 500μm; d-f: scale bar = 100μm).


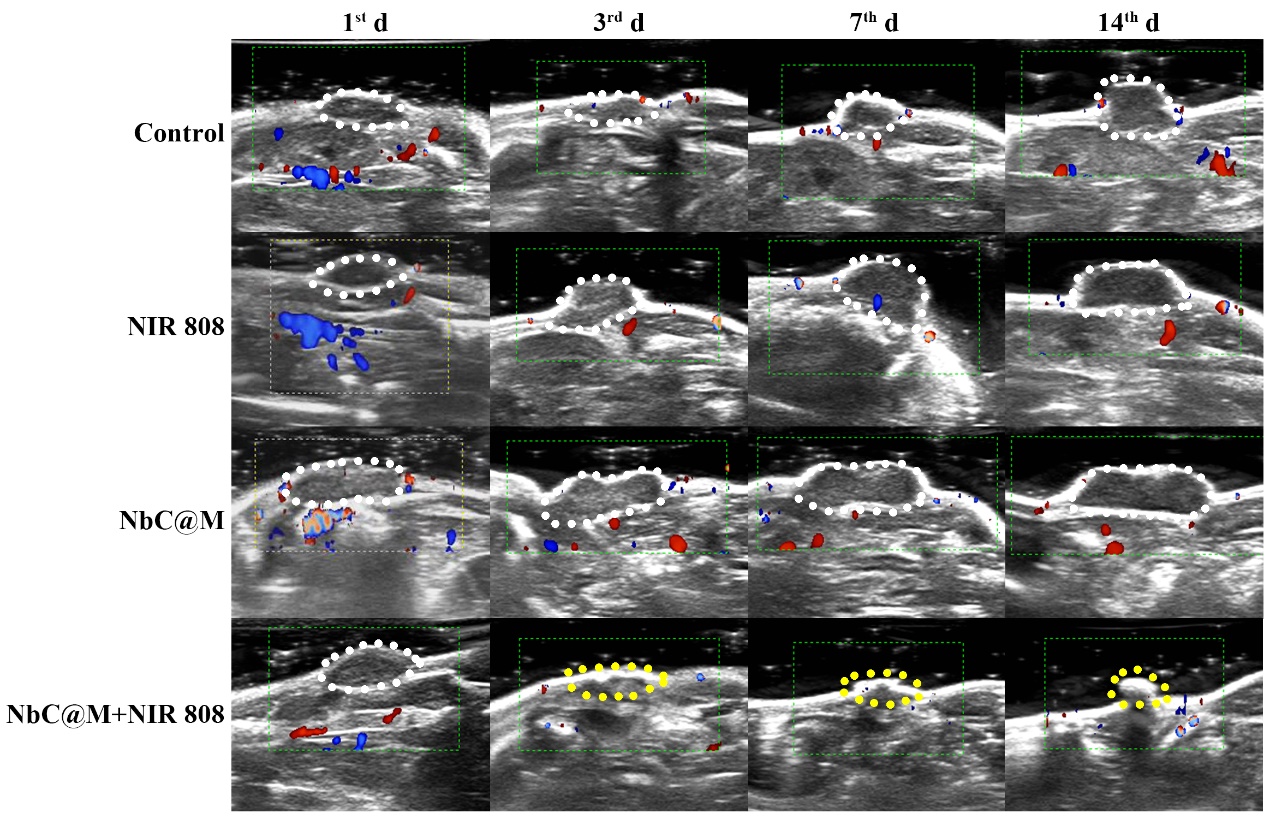


**Figure. S2** Color Doppler Flow Imaging (CDFI) observation for monitoring change process of solid tumor in mice before and after phototherapy. The dotted line indicated the tumor area site. It can be observed that the tumor tissue increases with time in the control, NIR808 and NbC@M group, while the tumor is cured to form scab (the yellow dotted line) in the NbC@M + NIR 808 group.


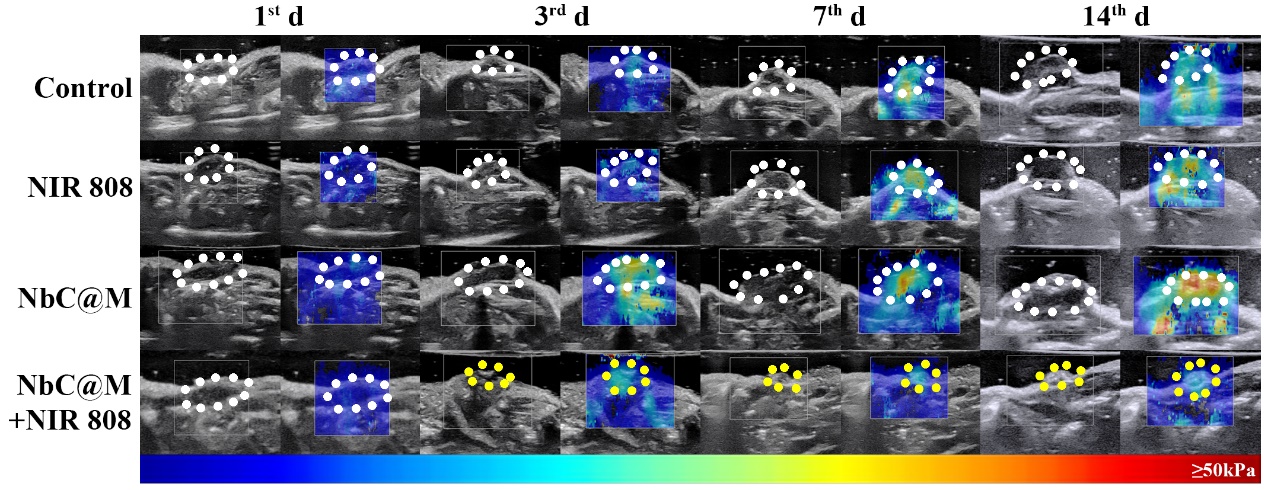


**Figure. S3** Shave wave elastography (SWE) observation for monitoring change process of solid tumor in mice before and after phototherapy. The dotted line indicated the tumor area site. The SWE value of tumor area is getting larger and larger, while the SWE value of scabby part is decreasing, indicating that the tumor area is getting better after treatment.


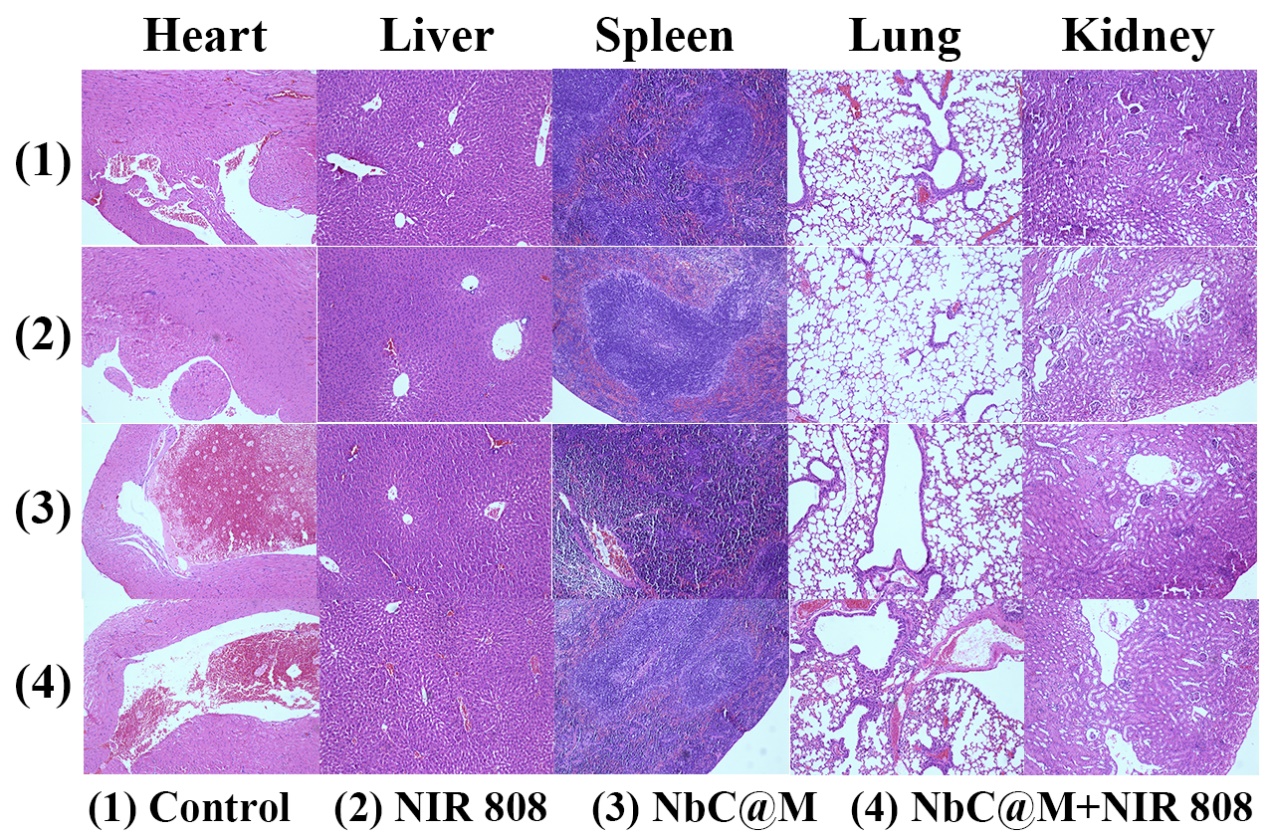


**Figure. S4** Histopathological analysis of mice major organs (heart, liver, spleen, lung, kidney) after different treatments (H&E, 10×10)


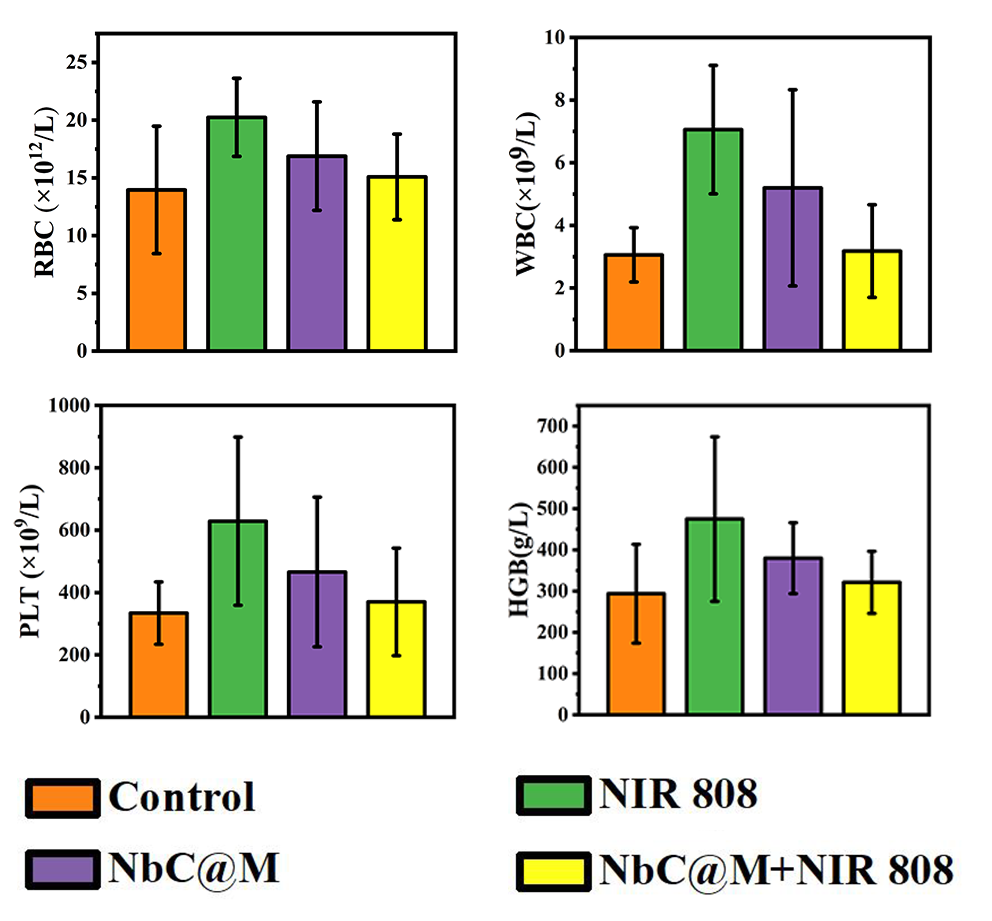


**Figure. S5** The blood biochemistry results of all the mice at 14^th^ day after different treatments.


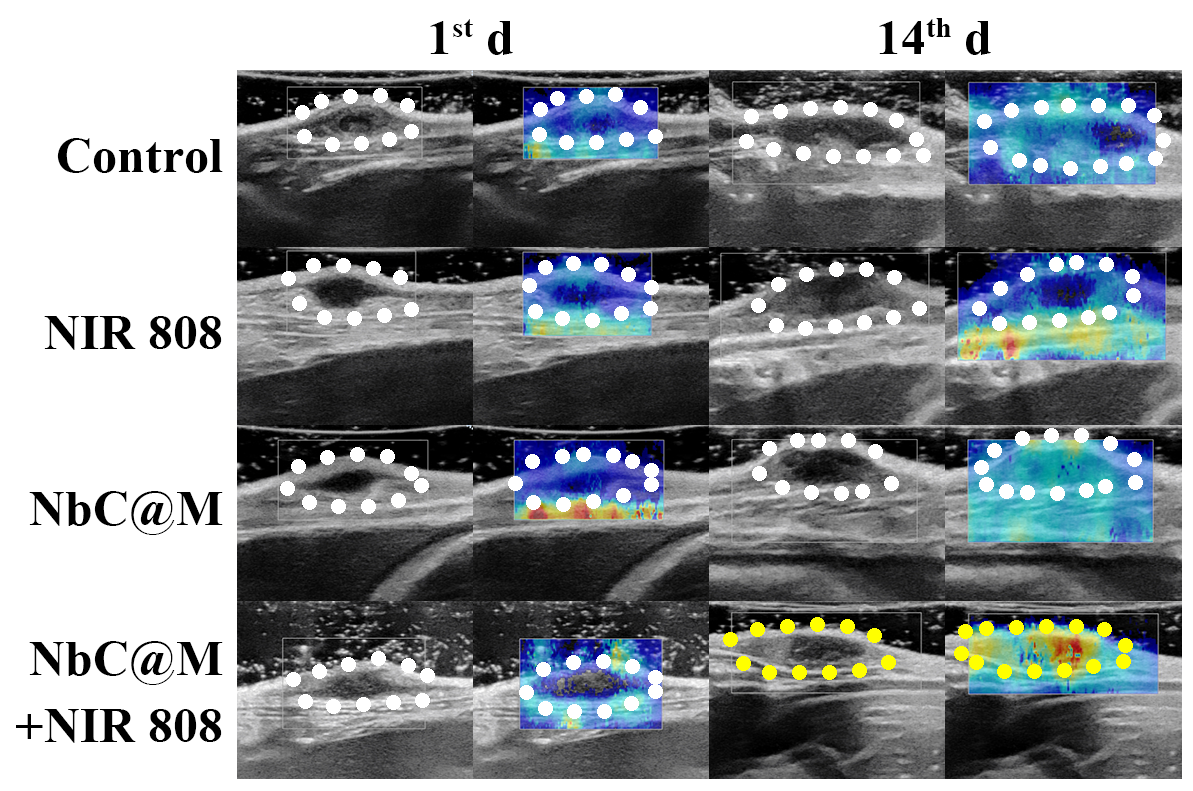


**Figure. S6** Shave wave elastography (SWE) observation for monitoring change process of solid tumor in rabbit before and after phototherapy. The dotted line indicated the tumor area site.


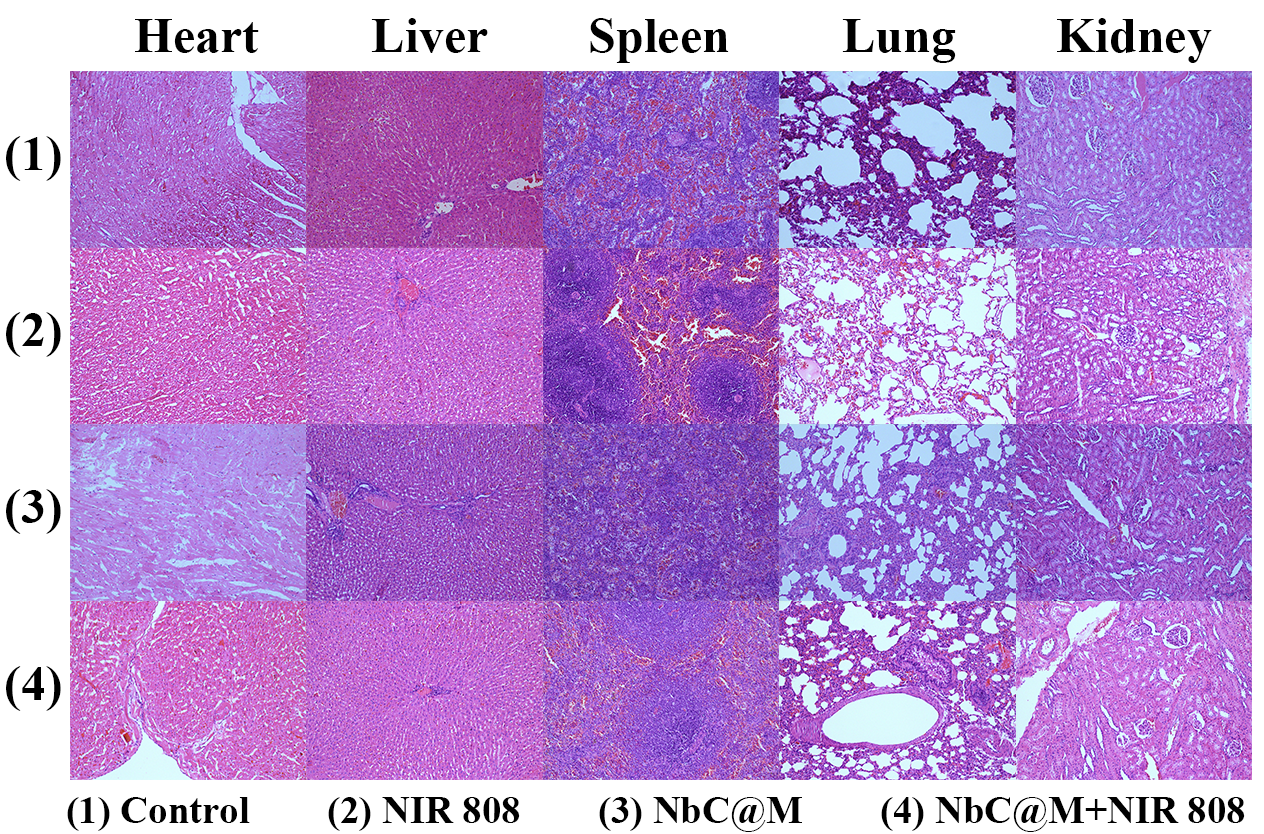


**Figure. S7** Histopathological analysis of rabbits’ major organs (heart, liver, spleen, lung, kidney) after different treatments (H&E, 10×10).
